# Use of Peer-Led Web-Based Platforms for Peer-Assisted Learning Among Canadian Anesthesia Residents and Fellows: Cross-Sectional Study

**DOI:** 10.2196/47977

**Published:** 2023-11-13

**Authors:** Casey Li, Maria Salman, Tariq Esmail, Clyde Matava

**Affiliations:** 1 Faculty of Medicine University of British Columbia Vancouver, ON Canada; 2 Department of Anesthesia and Pain Medicine Hospital for Sick Children Toronto, ON Canada; 3 Department of Anesthesiology and Pain Medicine Termerty Medicine University of Toronto Toronto, ON Canada; 4 Department of Anesthesiology and Pain Management University Health Network Toronto, ON Canada

**Keywords:** medical education, anesthesia, residents, fellowship, social media, peer led, peer assisted learning, anesthesiology, mobile device usage, health care, medical trainee, perception, mobile app, digital health

## Abstract

**Background:**

Peer-assisted learning (PAL) using peer-led web-based platforms (PWPs), including social media, can be a highly effective method of supporting medical trainees. PWPs, such as mobile apps for sharing anesthesia resources and social media groups or discussion forums pertaining to anesthesia training, may play a role in facilitating anesthesia trainee-led web-based education. However, there have been many challenges facing anesthesia trainees when it comes to incorporating PWPs, especially social media and mobile apps for PAL.

**Objective:**

The primary objective of this survey was to assess the proportion of trainees that use social media and mobile apps. The secondary objective was to identify the trainees’ perceptions on the use of social media and mobile apps for educational purposes, including PAL.

**Methods:**

This cross-sectional study was conducted through a survey administered via email at a single large academic center. The survey tool collected data between 2016 and 2017 on the following: demographic data (year of study, field of specialty), use of technology and web-based resources for medicine, use of social media platforms for anesthesia or training, benefits and barriers to future uses of social media for training, and ideas for trainee-led websites. Descriptive statistics were reported.

**Results:**

In total, 80 anesthesia trainees (51 residents and 29 fellows) responded to the survey (response rate of 33% of out 240 trainees contacted). All trainees reported having a mobile device that most (n=61, 76%) reported using multiple times a day to access medical resources. The highest perceived benefits of PWPs according to residents were that the most valuable information was available on-demand (n=27, 53%), they saved time (n=27, 53%), and they improved their overall learning experience within anesthesia (n=24, 47%). In comparison, fellows thought that PWPs were beneficial because they provided multiple perspectives of a single topic (n=13, 45%) and served as an additional platform to discuss ideas with peers (n=13, 45%). The most popular platforms used by both residents and fellows were Facebook (residents: n=44, 86%; fellows: n=26, 90%) followed by LinkedIn (residents: n=21, 42%; fellows: n=9, 29%). Even though most anesthesia trainees used social media for personal reasons, only 26% (n=21) reported having used resident- or fellow-driven PWP resources. Examples of PWPs that trainees used included anesthesia groups and a resident Dropbox resource folder.

**Conclusions:**

There was generally an acceptance for using PWPs for PAL as they provided various benefits for trainees at all levels of learning. PWPs have the potential to garner an increased sense of community and sharing within learning experiences throughout all levels of training. The information gained from this survey will help inform the basis for developing an anesthesia trainee-led e-learning platform.

## Introduction

The use of social media has become a fundamental part of everyday life in the modern world. As social media has become increasingly integrated into health care, it has become an essential tool for health care professionals, researchers, and patients to communicate, share information, and stay up-to-date [1]. For medical professionals, social media platforms provide an effective and efficient method of communicating, collaborating, and sharing knowledge [2,3]. In medical education, peer-assisted learning (PAL) can be a highly effective method of supporting medical trainees [4,5]. PAL refers to the process in which a group of peers who are not professional educators help each other learn and educate themselves [6,7]. Peer-led web-based platforms (PWPs), such as social media or mobile apps, play an important role in anesthesia education and may help support and advance PAL [8,9]. PAL is described here as an opportunity for both tutor and student to gain new knowledge from their shared experience.

The medical profession has been an ever-evolving field that requires ongoing education and training for medical professionals. Thus, the advantages of PWPs and PAL have been widely recognized within medicine. Specifically, medical trainees benefit from PAL as it allows them to develop their clinical skills, knowledge, and understanding of medical concepts and procedures. PWPs also allow medical trainees to access a wider range of resources and support from their peers through collaboration [9]. Additionally, they can be a powerful tool to engage younger trainees and encourage informal learning in a modern digital environment. Although PAL may happen in various settings, including in-person, recent technological advances have enabled learners to use social media to facilitate this type of learning [10]. Medical trainees frequently use web-based resources and mobile devices already [11,12], and social media’s infrastructure was built on the concept of facilitating community building. Significantly, residents also believe that social media plays a role in medical education [13,14]**.** With the rise of social media platforms, medical trainees have access to various sources for learning and collaboration. Social media allows medical trainees to share their knowledge and experiences with others, enabling them to learn from each other in a safe, supportive environment [15].

Having a wide net of support with seniors who have more knowledge and interacting with peers through a problem-based learning environment has been found to allow for residents to attain better academic achievements [16]. However, many medical learners describe medical training as isolating as it is challenging to connect with other residents due to limitations imposed by individual rotation schedules and rotations at different hospitals [17]. This problem has been exacerbated during the COVID-19 pandemic, where this type of learning has become especially important as there are limited traditional educational approaches available [15]. However, medical trainees still face many challenges when incorporating PWPs, including social media and mobile apps [18].

In this study, we explored the current use of social media and mobile apps as PWPs for PAL amongst medical trainees and their potential applications to facilitate the learning process. The primary objective of this survey was to assess the proportion of trainees that use social media. The secondary objectives of this survey were to identify trainees’ perceptions of using social media for educational purposes, evaluate the acceptability of a PWP, and identify how PWPs and PAL could be tailored to trainees in anesthesia.

## Methods

### Ethical Considerations

The study received ethics approval from the University of Toronto (RISPN33955).

### Setting

This study was conducted through a web-based cross-sectional survey design at a single large academic center, the University of Toronto’s Anesthesia program. The survey tool collected data between May 2016 and June 2016.

The survey was administered on the web using Qualtrics Experience Management software (Qualtrics). Eligible participants included all residents and fellows registered for training in the Department of Anesthesia during the study period. We sent out an email inviting all residents and fellows to participate in the survey. Approximately 2 weeks after the initial email, we sent a reminder email. The survey was accessible by computer or mobile device (Multimedia Appendix 1). Completion of the survey implied consent. There were no incentives offered for this study, and each participant received a unique link to prevent double entry.

### Participants

The survey was presented to all residents and fellows in the Anesthesia program. Respondents were assured that their participation was voluntary, would not impact their training as it was anonymous, and the information they provided would be reported in aggregate.

### Survey Design

Following a literature review on similar surveys, we designed the survey and pretested it on 3 volunteer residents [19-24]. Only minor changes were made following feedback. The responses from the testing phase were not included in the analysis.

The survey tool collected data on the following: demographic data (year of study, field of specialty); use of technology and web-based resources for medicine; use of social media, mobile apps and PAL for anesthesia or training; benefits and barriers to future uses of social media, mobile apps, and PAL for training; and ideas for trainee-led websites.

### Sample Size and Sampling

Previous survey research among anesthesiologists reported a median response rate of 37% (IQR 25%-46%) [25-31]. We aimed to achieve a similar response rate and invited all eligible fellows and residents.

### Outcomes Measured

The primary outcome measured was the proportion of anesthesia trainees that use social media and mobile apps. Secondary outcomes included the perceived benefits of PWPs, current or previous PWP resource use, and usage patterns of social media and PAL among anesthesia residents and fellows.

### Statistical Analysis

Descriptive statistics were used to summarize the results. Categorical variables were presented as frequency and proportion. The phrase “anesthesia trainees” was used when referring to both residents and fellows. The survey was reported according to the CHERRIES (Checklist for Reporting Results of Internet E-Surveys) checklist [29]. Statistical analyses were performed using JMP SAS software, version 9.4 (SAS Institute).

## Results

### Participant Demographics

A total of 240 anesthesia trainees (105 residents and 135 fellows) were eligible for the study. The response rate was 33% (n=80) with 49% (n=51) of anesthesia residents responding and 21% (n=29) of anesthesia fellows responding. Table 1 shows the respondents’ demographics.

**Table 1:** Participant (n=80) demographics.

Training		Participants, n (%)
**Residents (n=51)**		
	PGY^a^-1	16 (31)
	PGY-2	10 (20)
	PGY-3	9 (17)
	PGY-4	7 (15)
	PGY-5	9 (17)
**Fellows (n=29)**		
	Regional	6 (20)
	Ambulatory regional	1 (3)
	Obstetrics	4 (14)
	Cardiac	1 (3)
	Education and simulation	1 (3)
	Chronic pain	3 (10)
	Hepatobiliary and transplantation	2 (8)
	Trauma and critical care	1 (3)
	Trauma and neuroanesthesia	2 (8)
	Neuroanesthesia	3 (10)
	Perioperative	3 (10)
	Unspecified	2 (8)

^a^PGY: postgraduate year.

### Use of Technology for Medical Education

Table 2 shows the devices used by anesthesia residents and fellows for medical education. The most commonly used device to access web-based anesthesia materials was an Apple product, including MacBooks (n=51, 64%), iPhones (n=46, 58%), and iPads (n=35, 44%). On the other hand, fewer trainees used Microsoft Windows computers (n=29, 36%), Android phones (n=13, 16%), or Android tablets (n=4, 5%). All trainees reported having a mobile device.

Most medical trainees accessed medical resources with the help of their mobile device multiple times daily (n=61, 76%). On average, each trainee had 5 (SD 3) medical- or anesthesia-related apps downloaded on any of their devices. Generally, each trainee had 1-5 apps downloaded on their phone, but 1 fellow possessed 31-50 apps. Additionally, 10 residents had no medical software apps downloaded.

Further examination revealed that residents and fellows used their devices primarily for different objectives. For example, while both anesthesia residents and fellows used their devices to find drug information (residents: n=47, 92%; fellows: n=23, 93%), residents mainly took advantage of devices to read point of care information (n=45, 88%) and use cloud-based storage (n=43, 84%). In comparison, fellows made use of devices to search for journal articles (n=28, 97%), read journal articles (n=27, 93%), and find practice guidelines (n=27, 93%). These findings suggest that residents may have used devices to acquire new knowledge, unlike fellows, who used devices to stay informed of new practices.

**Table 2:** Technology use for medical education purposes.

Technology use		Residents (n=51), n (%)	Fellows (n=29), n (%)	All trainees (n=80), n (%)
**Devices used for anesthesia**				
	Macbook	34 (67)	17 (59)	51 (64)
	iPhone or iPod	31 (61)	15 (52)	46 (58)
	iPad	22 (43)	13 (45)	35 (44)
	Windows computer	16 (31)	13 (45)	29 (36)
	Android phone	8 (16)	5 (17)	13 (16)
	Android tablet	2 (4)	2 (7)	4 (5)
	Other devices	1 (2)	0 (0)	1 (1)
	No device	0 (0)	0 (0)	0 (0)
**Purposes for device use**				
	Find drug information	47 (92)	27 (93)	74 (93)
	Find practice guidelines	42 (82)	27 (93)	69 (86)
	Point of care information	45 (88)	24 (83)	69 (86)
	Cloud-based collaborative storage	43 (84)	24 (83)	67 (84)
	Retrieve journal articles	37 (73)	28 (97)	65 (81)
	Read journal articles	37 (73)	27 (93)	64 (80)
	Clinical calculations	40 (78)	24 (83)	64 (80)
	Call schedule via Excel sheet	42 (82)	22 (76)	64 (80)
	Call schedule via device calendar	35 (69)	24 (83)	59 (74)
	Facebook	37 (73)	22 (76)	59 (74)
	Checked online evaluation system	37 (73)	13 (45)	50 (63)
	Take notes	34 (67)	15 (52)	49 (61)
	Time-off manager	25 (49)	1 (3)	26 (33)
	Twitter	12 (24)	5 (17)	17 (21)
	Blackboard course portal	8 (16)	1 (3)	9 (11)
**Frequency of device use for medical purposes**				
	More than once per day	39 (77)	22 (76)	61 (76)
	Once per day	7 (14)	5 (17)	12 (15)
	Several times a week	5 (10)	2 (7)	7 (9)
	Less than several times a week	0 (0)	0 (0)	0 (0)
**Number of medical apps on devices**				
	0	10 (20)	0 (0)	10 (13)
	1-5	27 (53)	15 (52)	42 (53)
	6-10	12 (24)	12 (41)	24 (30)
	11-20	2 (4)	0 (0)	2 (3)
	21-30	0 (0)	1 (3)	1 (1)
	31-50	0 (0)	1 (3)	1 (1)
	>50	0 (0)	0 (0)	0 (0)

### Use of Social Media by Trainees

All but 4 anesthesia trainees reported using social media platforms. The most popular platform that residents and fellows used was Facebook (residents: n=43, 86%; fellows: n=25, 89%), as shown in Table 3. In addition, a popular platform used by residents was LinkedIn (n=21, 42%), while this was only used by 8 (29%) fellows. Most trainees reported using social media for anesthesia infrequently as they either mainly followed people rather than posted or posted less than once every month (n=59, 74%).

**Table 3:** Social media usage.

Usage			Residents (n=51), n (%)		Fellows (n=29), n (%)		All trainees (n=80), n (%)
**Social media platforms used**							
	Facebook	43 (86)		25 (89)		68 (87)	
	LinkedIn	21 (42)		8 (29)		29 (37)	
	QxRead	14 (28)		5 (18)		19 (24)	
	Twitter	12 (24)		5 (18)		17 (22)	
	ResearchGate	12 (24)		5 (18)		17 (22)	
	Mendeley	5 (10)		7 (25)		12 (15)	
	Pinterest	5 (10)		4 (14)		9 (12)	
	Academia	1 (2)		3 (11)		4 (5)	
	None	3 (6)		1 (4)		4 (5)	
**Social media usage for anesthesia**							
	One post per day	1 (2)		2 (7)		3 (4)	
	One post per week	1 (2)		1 (3)		2 (3)	
	One post per month or less	6 (12)		3 (10)		9 (11)	
	Only follow others	31 (61)		19 (66)		50 (63)	
	No social media	12 (24)		4 (14)		16 (20)	

### Use of PWPs for Anesthesia

Only 26% (n=21) of respondents reported using resident- or fellow-driven PWP resources (Table 4). Instead, trainees brought up resources, such as anesthesia groups and a resident Dropbox resource folder, as examples of PAL that they currently use.

Residents and fellows agreed that a PWP’s perceived benefits were useful for answering questions (n=38, 46%). However, their views diverged in relation to other potential benefits. The highest perceived benefits of PAL according to residents included that the most valuable information was available on-demand (n=27, 53%), that it saved time (n=27, 53%), and that it improved their overall learning experience within anesthesia (n=24, 47%). On the other hand, fellows noted the benefits of PAL being that it helped them appreciate various perspectives of a single topic (n=13, 45%), provided an additional platform to discuss things with peers (n=13, 45%), and kept them up-to-date with course announcements (n=13, 45%). PAL also enabled more accessible communication between peers and supervisors (n=13, 45%). Therefore, PWPs may be beneficial for residents and fellows, but the benefits may differ depending on the level of training.

**Table 4:** Peer-led web-based platform (PWP) usage.

Usage		Residents (n=51), n (%)	Fellows (n=29), n (%)	All trainees (n=80), n (%)
**Perceived benefits of PWPs**				
	Receive answers to questions	23 (45)	14 (48)	38 (46)
	Saves time	27 (53)	8 (28)	35 (44)
	Information is easily accessible	27 (53)	7 (24)	34 (43)
	Improves learning experience	24 (47)	9 (31)	33 (41)
	Stay up-to-date	19 (37)	13 (45)	32 (40)
	Provides useful external resources	21 (41)	11 (38)	32 (40)
	Alternate points of view of topics	18 (35)	13 (45)	31 (39)
	Peer discussions	18 (35)	13 (45)	31 (39)
	Channel for communication	17 (33)	13 (45)	30 (38)
	Motivational	21 (41)	8 (28)	29 (36)
	Sense of community	15 (29)	13 (45)	28 (35)
	Enhance understanding of materials	19 (37)	6 (21)	25 (31)
	Improve connectedness within program	18 (35)	6 (21)	24 (30)
	Highlights important learning concepts	14 (28)	5 (17)	19 (24)
	Helps advance career	9 (18)	7 (24)	16 (20)
	Encourages reflection	7 (14)	7 (24)	14 (18)
	Enjoy personally contributing	5 (10)	1 (3)	6 (8)
	No benefits	4 (8)	1 (3)	5 (6)
**PWP resource use (current or previous)**				
	Yes	16 (31)	5 (17)	21 (26)
	No	35 (69)	24 (83)	59 (74)

### Future Uses

Table 5 demonstrates considerations for a PWP. The amount of money that anesthesia trainees reported being willing to spend for access to a PWP fluctuated from nothing to >CAD $50 (US $36; a currency exchange rate of CAD $1=US $0.72 was applicable). More residents indicated that they would spend less than CAD $10 (US $7.20) per year (n=28, 55%) compared to those who would spend more than CAD $10 (US $7.20) per year (n=23, 45%). Comparatively, more fellows indicated their willingness to spend over CAD $10 (US $7.20) per year on medical apps (n=17, 59%).

Most trainees (n=71, 89%) would consider using a secure platform to access contact information for all residents or fellows in the Anesthesia program. Similarly, most trainees (n=69, 86%) indicated that they would be open to committing at least 1 hour to creating a resource on residency electives and fellowship experience to assist future trainees. On the other hand, most fellows (n=22, 76%) indicated that they would consider writing reflective or educational blog articles to share with other trainees, while only relatively few residents (n=19, 37%) were interested in the idea. In addition, residents and fellows both demonstrated a large interest in a web-based platform that allows residents and fellows to share documents (residents: n=46, 90%; fellows: n=27, 93%). Most trainees preferred a web-based platform where they can easily access peers’ contact information, share resources on electives and experiences, and share documents.

However, barriers that trainees considered included that they felt there may be poor engagement (n=34, 43%), that resources may lack in quality (n=18, 23%), and that they may not have time (n=7, 9%). Few trainees noted concerns about privacy issues (n=3, 4%), and many indicated that they did not see any potential barriers (n=18, 23%) to a peer-led site.

**Table 5:** Implementation of a peer-led web-based platform (PWP).

Implementation considerations		Residents (n=51), n (%)	Fellows (n=29), n (%)	All trainees (n=80), n (%)
**Amount willing to spend for a PWP application, CAD $ (US $)^a^**				
	Nothing	9 (18)	1 (3)	10 (13)
	<1 (0.72)	0 (0)	2 (7)	2 (3)
	1 (0.72)-5 (3.60)	9 (18)	3 (10)	12 (15)
	5 (3.60)-10 (7.20)	10 (20)	6 (21)	16 (20)
	11 (7.92)-20 (14.40)	6 (12)	10 (35)	16 (20)
	21 (15.12)-30 (21.60)	8 (16)	0 (0)	8 (10)
	31 (21.60)-50 (36)	4 (8)	5 (17)	9 (11)
	>50 (36)	5 (10)	2 (7)	7 (9)
**Likelihood of accessing peer contact information**				
	Not likely	2 (4)	1 (3)	3 (4)
	Somewhat unlikely	6 (12)	0 (0)	6 (8)
	Maybe	13 (26)	12 (41)	25 (31)
	Somewhat likely	15 (29)	8 (28)	23 (29)
	Very likely	15 (29)	8 (28)	23 (29)
**Likelihood of contributing to a resident or fellow guidebook**				
	Not likely	4 (8)	1 (3)	5 (6)
	Somewhat unlikely	5 (10)	1 (3)	6 (8)
	Maybe	13 (26)	12 (41)	25 (31)
	Somewhat likely	14 (28)	8 (28)	22 (28)
	Very likely	15 (29)	7 (24)	22 (28)
**Likelihood to write forum articles for fellow peers**				
	Not likely	19 (37)	3 (10)	22 (28)
	Somewhat unlikely	13 (26)	4 (14)	17 (21)
	Maybe	10 (20)	13 (45)	23 (29)
	Somewhat likely	5 (10)	8 (28)	13 (16)
	Very likely	4 (8)	1 (3)	5 (6)
**Likelihood to share notes or documents**				
	Not likely	3 (6)	2 (7)	5 (6)
	Somewhat unlikely	2 (4)	0 (0)	2 (3)
	Maybe	4 (8)	5 (17)	9 (11)
	Somewhat likely	10 (20)	15 (52)	25 (31)
	Very likely	32 (63)	7 (24)	39 (49)
**Barriers to a PWP site**				
	Poor engagement from peers	19 (37)	15 (52)	34 (43)
	Poor resource quality	13 (26)	5 (17)	18 (23)
	No time	4 (8)	3 (10)	7 (9)
	Privacy concerns	3 (6)	0 (0)	3 (4)
	No barriers	12 (24)	6 (21)	18 (23)

^a^A currency exchange rate of CAD $1=US $0.72 was applicable.

## Discussion

### Principal Findings

This study demonstrated that most anesthesia trainees use social media, especially Facebook. However, most trainees used social media for anesthesia-related purposes mainly to follow others, rather than to post on the platforms. In addition, all anesthesia trainees had access to personal electronic devices and the majority used them multiple times a day to access medical knowledge or discussions. However, trainees used these devices to achieve different goals; for example, residents mainly used them to find point of care information, while fellows found devices most helpful for reviewing journal articles and staying informed of new practices. Both levels of trainees described benefits of PALs but their perceived benefits may differ depending on the trainee’s level of training.

These findings suggest that, in an academic context, anesthesia trainees used social media mainly to receive educational information and stay informed. The findings from this study were consistent with previous research that indicated that social media use was common among medical trainees. Avcı et al [32] found that 93% of medical students used social media and 89% used it for professional purposes. Similarly, Bosslet et al [33] found that 94% of medical trainees used social media, with Facebook as the most common platform. More recently, Nisar et al [34] reported that 89% of students used social media and 69% used it for learning. However, in contrast to older studies, this study demonstrated that newer social media platforms, such as YouTube, Blackboard, WhatsApp, Twitter, Zoom, and Google+, were more popular than platforms like Facebook and LinkedIn. This may be related to the shifting popularity of social media platforms as technology evolves. Among anesthesia residents, a novel and bespoke multiapplication platform called DansMaBlouse was reported by users to improve point of care access to information, and the platform was noted to be easy to use [9]. These studies demonstrate that anesthesia trainees are willing to adopt emerging social media platforms for PAL.

PWPs were identified as a useful resource for anesthesia trainees as they allowed learners to easily ask questions and identify high-yield information, remain informed on beneficial resources, obtain a better learning experience, and feel connected to their class and community. These benefits may be accomplished through features such as discussion forums, cloud-based shared storage drives containing learning resources for anesthesia, feedback and communication channels for staff and learner communication, and social features such as group chats and status updates. The findings from this study were consistent with other studies that also demonstrated PAL to be effective for acquiring new knowledge and skills. Jauregui et al [35] also reported that medical trainees found that peer-guided simulations helped them expand their knowledge base, feel more confident in their skills, and improve their learning experience. Sriwigati and Musharyanti [36] also described that PAL increased health care students’ knowledge and confidence. Although PWPs were identified in our study to be useful amongst anesthesia trainees, they must be further developed and tailored according to training level as trainees’ goals differ as they progress through their education. For instance, although residents were more likely to share notes between each other, fellows were more likely to write reflective or educational articles for peers. To our knowledge, no other studies have directly examined how the goals of trainees differ depending on the level of training. Perceived barriers to implementing an anesthesia PWP included concerns regarding poor peer engagement and poor resource quality. However, in contrast, Jawhari et al [37] found that the top barriers to PAL in Saudi Arabia included taking more time than traditional teaching and peers having insufficient knowledge to teach. Very few people reported time concerns in our study, which may reflect differences in teaching methods in various countries. The DansMaBlouse platform is an example of a bespoke platform, the likes of which require development, financial resourcing, and relevant expertise; however, these resources may be out of reach for most trainees [9].

This study was conducted several years ago, so one of the limitations we encountered was that this study has only looked at traditional social media platforms, such as Facebook and Twitter (subsequently rebranded X), but over time these platforms may become outdated or their patterns of usage may change to involve other platforms, including WhatsApp and curated multiplatform resources [38-46]. Therefore, the study data may be limited in its utility for implementation into a new PAL-supporting PWP. Even though some of the traditional social media platforms have been around for a long time, there has been a recent shift toward newer platforms that offer different features, like video chatting and interactive chat rooms, including Zoom. This makes it easier for medical trainees to collaborate with each other and access up-to-date information. In addition, this study only reflected on a single-center experience. Each center may differ in trainee experience and goals, which may result in different PAL usage patterns. Thus, this study may not be generalizable to other centers or locations.

As technology and social media platforms continue to evolve, so too should the tools and platforms for PAL used by medical trainees. Currently popular platforms, such as TikTok, Slack, and Teams, offer trainees more information, improved communication (eg, through video chat and remote collaborations), and web-based discussion forums, which can all greatly benefit trainees in their learning. Additionally, as social media platforms become increasingly popular, these platforms can also be used to stay connected, share resources, collaborate with peers, and stay up-to-date with the latest course announcements. In doing so, medical trainees can build a strong network of peers to support their learning journey. Additionally, these platforms can provide trainees with access to valuable information on-demand, which can help save time and improve their overall learning experience within anesthesia.

### Conclusion

In conclusion, this survey examined the uses of technological devices, patterns of social media use, and the benefits and limitations of PAL to contribute to an anesthesia trainee’s experience. Our results suggested that trainees all have access to devices to use regularly for medical knowledge and that there has generally been an acceptance of using PAL for learning if it addressed concepts that are meaningfully individualized for the trainee’s level of education. While the benefit of PAL for building the anesthesia trainee community is unknown, PAL has the potential to garner an increased sense of community and sharing within learning experiences throughout all levels of training. The information gained from this survey may help inform the basis for developing an anesthesia trainee-led e-learning platform. Therefore, the opinions and information shared by participants within trainee programs could help directly influence the development of a pilot platform.

## Appendix

Multimedia Appendix 1 Survey tool.

## Abbreviations

CHERRIES: Checklist for Reporting Results of Internet E-Surveys
PAL: peer-assisted learning
PWP: peer-led web-based platform

## Data Availability

The data sets generated or analyzed during this study are available from the corresponding author on reasonable request.
