# Supplementary material for: Use of Peer-Led Web-Based Platforms for Peer-Assisted Learning Among Canadian Anesthesia Residents and Fellows: Cross-Sectional Study
Source: JMIR Form Res. 2023 Nov 13;7:e47977. doi: 10.2196/47977 (PMC10682924; doi:10.2196/47977)
Supplement: Multimedia Appendix 1 [file formative_v7i1e47977_app1.pdf]

Survey Of Attitudes Towards A Peer Led And Driven Online Resource For Anesthesia Trainees

Q1 This survey is only intended for information gathering to better design and facilitate components of a new resident sharing online platform for a way in which the residents of all years will engage and participate. Should the results be of significant findings, they may be used for future research purposes with REB approval. Your responses remain anonymous and not linked towards any component of evaluation.

Q2 What is your level of training?

- PGY 1 (1)
- PGY 2 (2)
- PGY 3 (3)
- PGY 4 (4)
- PGY 5 (5)
- Fellow Year 1 (6)
- Fellow Year 2 (7)
- Fellow Year 3 (8)

Display This Question:

If What is your level of training? Fellow Year 1 Is Selected

And What is your level of training? Fellow Year 2 Is Selected

And What is your level of training? Fellow Year 3 Is Selected

Q3 What is your fellowship?

- Regional (1)
- Obstetrics (2)
- Cardiac (3)
- Advanced Airway Mgmt (4)
- Education & Simulation (5)
- Chronic Pain (6)
- Thoracic (7)
- Hepatobiliary & Transplantation (8)
- Trauma & Critical Care (9)
- Trauma & Neuroanesthesia (10)
- Neuroanesthesua (11)
- Perioperative (12)
- Ambulatory Regional (13)

Q4 Which device(s) do you most frequently use to access ANESTHESIA related online material (select all that apply)

- ❑ a. Mac (1)
- ❑ b. Windows (2)
- ❑ c. Iphone/ipod touch (3)
- ❑ d. Ipad (4)
- ❑ e. Android phone (5)
- ❑ f. Android tablet (6)
- ❑ g. Blackberry (7)
- ❑ h. Other device (8)

Q5 Have you used your online resources (laptop/mobile) for any of the following purposes? (check all that apply)

- ❑ I don't own a mobile device (1)
- ❑ Take notes (2)
- ❑ Find drug information (3)
- ❑ Find practice guidelines (4)
- ❑ Read point of care info (ie. Up to date, dynamed etc..) (5)
- ❑ Clinical calculations (6)
- ❑ Search for journal articles (7)
- ❑ Read journal articles (8)
- ❑ Access your call schedule calendar (in excel/spreadsheet) (9)
- ❑ Access your call schedule in an integrated calendar on your device (10)
- ❑ Used dropbox, goole drive, MEGA, cloud based storage (11)
- ❑ Used Blackboard app (12)
- ❑ Twitter (13)
- ❑ Facebook (14)
- ❑ Checked POWER (15)
- ❑ Time off Manager (16)
- ❑ To access Mendeley/Researchgate/Academia (17)

Q6 How often do you use your mobile device to access medical resources? (medical resource means both downloaded application as well as websites that allow you to access them through the web browser on your device)

- ❍ a. More than once a day (1)
- ❍ b. Once a day (2)
- ❍ c. Several times a week (3)
- ❍ d. Several times a month (4)
- ❍ e. Once a month (5)
- ❍ f. Less than once a month (6)
- ❍ g. Never (7)

Q7 Which of following do you perceive as benefits for a peer-led, resident/fellow driven online sharing platform?

- ❑ I don't see any benefits (1)
- ❑ Betters my overall learning experience of anesthesia (2)
- ❑ Helps to improve my understanding of the anesthesia curriculum material (3)
- ❑ Motivates me to study (4)
- ❑ Helps to look at certain topics in a different context (5)
- ❑ I will find it useful to discuss things with my peer group online (6)
- ❑ I often find resources and web links useful towards my studies (7)
- ❑ I will feel like a part of a community (8)
- ❑ I will feel like my opinions are valued online (9)
- ❑ The information I REALLY need is readily accessible on demand (10)
- ❑ Useful for answering my questions (11)
- ❑ Good for reflecting on professional/legal/ethical issues (12)
- ❑ Easy to communicate with my peers and supervisors (13)
- ❑ Keeps me up to date with the latest course announcements and medical related news (14)
- ❑ A useful tool for advancing my career (15)
- ❑ Will help me focus my learning on important concepts (16)
- ❑ Will allow me to save time by access content shared by residents (17)
- ❑ Will increase the sense of connectedness in anesthesia residency (18)

Q8 Have you previously or do you currently use any peer-led, resident/fellow driven online resource?

- ❍ Yes (1)
- ❍ No (2)

Q9 If you currently use a peer-led, resident/fellow driven online resource, please name it/them.

Q10 How many anesthesia/medical apps do you have on your device(s)?

- ❍ 0 (1)
- ❍ 1-5 (2)
- ❍ 6-10 (3)
- ❍ 11-20 (4)
- ❍ 21-30 (5)
- ❍ 31-50 (6)
- ❍ >50 (7)

Q11 How much would you be willing to pay for an anesthesia/medical apps per year?

- ❍ Nothing (1)
- ❍ <$1 (2)
- ❍ $1-5 (3)
- ❍ $5-10 (4)
- ❍ $11-20 (5)
- ❍ $21-30 (6)
- ❍ $31-50 (7)
- ❍ >$50 (8)

Q12 Which of the following social media platform(s) do you use? (Check all that apply)

- ❑ Twitter (1)
- ❑ Facebook (2)
- ❑ QxRead (3)
- ❑ Researchgate (4)
- ❑ LinkedIn (5)
- ❑ Mendeley (6)
- ❑ Academia (7)
- ❑ Pinterest (8)
- ❑ None (9)

Q13 Rate your use of social media for Anesthesia? *

- ❍ a. Frequent – I post about one a day (1)
- ❍ b. Frequent – I post about once a week (2)
- ❍ c. Infrequent – I post about once a month or less (3)
- ❍ d. Infrequent – I tend to only follow people (4)
- ❍ I don't have / use social media (5)

Q14 Would you be interested in linking your social media (e.g twitter) to a site where residents or fellows only could see your tweets in a feed?

- ❍ Yes (1)
- ❍ Maybe (2)
- ❍ No (3)

Display This Question:

If What is your Level of Training? PGY 1 Is Selected
And What is your Level of Training? PGY 2 Is Selected
And What is your Level of Training? PGY 3 Is Selected
And What is your Level of Training? PGY 4 Is Selected
And What is your Level of Training? PGY 5 Is Selected

Q15 Do you know there is a Blackboard Portal for U of T?

- ❍ Yes (1)
- ❍ Maybe (2)
- ❍ No (3)

Display This Question:

If What is your Level of Training? PGY 1 Is Selected
And What is your Level of Training? PGY 2 Is Selected
And What is your Level of Training? PGY 3 Is Selected
And What is your Level of Training? PGY 4 Is Selected
And What is your Level of Training? PGY 5 Is Selected

Q16 How often do you check your personal Blackboard on a computer or through the mobile app?

- ❍ Yes – every day (1)
- ❍ Yes – once or twice (2)
- ❍ No – signed on once and haven’t since (3)
- ❍ No – I have never tried (4)

Q17 How likely would you use an online platform to access contact information for all residents in the current program (PGY1-5) or all the fellows in current training. (ie. a 'log in' protected website with contact information)

- 1 - Not Likely (1)
- 2 - Somewhat unlikely (2)
- 3 - Maybe (3)
- 4 - Somewhat likely (4)
- 5 - Very likely (5)

Q18 How likely would you be to commit to contributing to an 'elective catalogues' or 'fellowship experience' resource when finishing a rotation? (Time commitment of appx. 1 hour)

- 1 - Not likely (1)
- 2 - Somewhat unlikely (2)
- 3 - Maybe (3)
- 4 - Somewhat likely (4)
- 5 - Very likely (5)

Q19 What do you consider as barriers to accessing a proposed resident or fellow led site?

- I don't have time (1)
- Privacy issues (2)
- Quality of the resource (3)
- Resident participation (4)
- I don't see any barriers (5)

Q20 How likely are you to use a forum to post articles written by you/ "blog" like, in various categories to be shared with other residents or fellows (educational and/or reflective)?

- 1 - Not Likely (1)
- 2 - Somewhat unlikely (2)
- 3 - Maybe (3)
- 4 - Somewhat Likely (4)
- 5 - Very likely (5)

Q21 How likely would you use an online platform to share documents between all residents (PGY1-5) or fellows (ie. Like a shared dropbox or google drive for all in your training [residency or fellowship])

- 1 - Not Likley (1)
- 2 - Somewhat Unlikely (2)
- 3 - Maybe (3)
- 4 - Somewhat Likely (4)
- 5 - Very Likely (5)

Display This Question:

If What is your level of training? PGY 1 Is Selected
And What is your level of training? PGY 2 Is Selected
And What is your level of training? PGY 3 Is Selected
And What is your level of training? PGY 4 Is Selected
And What is your level of training? PGY 5 Is Selected

Q22 What else would you like to see in a resident focused website for Anesthesia at U of T? (tell us everything you want)

Display This Question:

If What is your Level of Training? PGY 1 Is Selected

And What is your Level of Training? PGY 2 Is Selected

And What is your Level of Training? PGY 3 Is Selected

And What is your Level of Training? PGY 4 Is Selected

And What is your Level of Training? PGY 5 Is Selected

Q23 What would you suggest the URL for a resident anesthesia website be: (shorter is better, eg. Radiology has www.radlink.ca) - any and all guesses welcome
